# A nationwide study on new onset atrial fibrillation risk factors and its association with hospital mortality in sepsis patients

**DOI:** 10.1038/s41598-024-62630-x

**Published:** 2024-05-28

**Authors:** Yi-wei Liu, Yi-fan Wang, Yan Chen, Run Dong, Shan Li, Jin-min Peng, Rong Liufu, Li Weng, Yang Xu, Bin Du

**Affiliations:** 1 Department of Pharmacy Administration and Clinical Pharmacy, School of Pharmaceutical Sciences, Peking University Health Science Center, No. 38 Xueyuan Road, Beijing, 100191 China; 2 Medical Intensive Care Unit, State Key Laboratory of Complex Severe and Rare Diseases, Peking Union Medical College Hospital, Peking Union Medical College, Chinese Academy of Medical Sciences, Beijing, 100730 China; 3 Department of Medical Epidemiology and Biostatistics (MEB), Karolinska Institutet, Stockholm, Sweden

**Keywords:** Sepsis, Atrial fibrillation, Risk factor, Cohort study, Risk factors, Disease prevention, Public health

## Abstract

Atrial fibrillation (AF) is the most common arrhythmia and its incidence increases with sepsis. However, data on new-onset AF during sepsis hospitalization remain limited in China. We aimed to evaluate the incidence, risk factors, and associated mortality of new-onset AF in sepsis patients in China. We conducted a retrospective study using the National Data Center for Medical Service system, from 1923 tertiary and 2363 secondary hospitals from 31 provinces in China from 2017 to 2019.In total we included 1,425,055 sepsis patients ≥ 18 years without prior AF. The incidence of new-onset AF was 1.49%. Older age, male sex, hypertension, heart failure, coronary disease, valvular disease, and mechanical ventilation were independent risk factor. New-onset AF was associated with a slight increased risk of mortality (adjusted RR 1.03, 95% CI 1.01–1.06). Population attributable fraction suggested AF accounted for 0.2% of sepsis deaths. In this large nationwide cohort, new-onset AF occurred in 1.49% of sepsis admissions and was associated with a small mortality increase. Further research should examine whether optimized AF management can improve sepsis outcomes in China.

## Introduction

### Background

Atrial fibrillation (AF) is the most common arrhythmia encountered in clinical practice, affecting over 30 million individuals worldwide^1^. The presence of AF is associated with increased risks of stroke, heart failure, dementia, and mortality^2^. Sepsis is a life-threatening organ dysfunction caused by a dysregulated host response to infection^3^. Septic patients are prone to develop new-onset AF during their intensive care unit (ICU) stay, with the reported incidence ranging from 5 to 42%^4,5^.

The development of new-onset AF in septic patients poses significant challenges in their management. AF can lead to rapid ventricular response and hemodynamic instability, which can be detrimental in critically ill patients^6^. Existing evidence also suggests an association between new-onset AF and worse clinical outcomes in sepsis. In a systematic review and meta-analysis of 13 studies in high-income countries, new-onset AF was associated with a 1.69-fold increased risk in hospital mortality in septic patients^7^. However, few evidence was available for low-and-middle income countries. Several mechanisms have been proposed to explain the predilection of sepsis patients to develop new-onset AF, including systemic inflammation, neurohormonal activation, electrolyte disturbances, and myocardial dysfunction^8^. However, the risk factors that specifically predispose sepsis patients to new-onset AF are not completely understood. Kuipers and colleagues reported that diabetes reduced risk for new-onset AF incidence in sepsis patients^9^.

### Objective

Elucidating the risk factors of new-onset AF among sepsis patients can help identify those at high risk of developing this arrhythmia. This may allow targeted monitoring or prophylactic interventions. Moreover, a better understanding of the relationship between new-onset AF and mortality in this population can aid prognostication and guide management decisions. In this study, we aim to determine the risk factors for new-onset AF and evaluate its impact on hospital mortality in patients with sepsis based on national-wide claims data.

## Results

### Baseline characteristics

**Figure 1:**
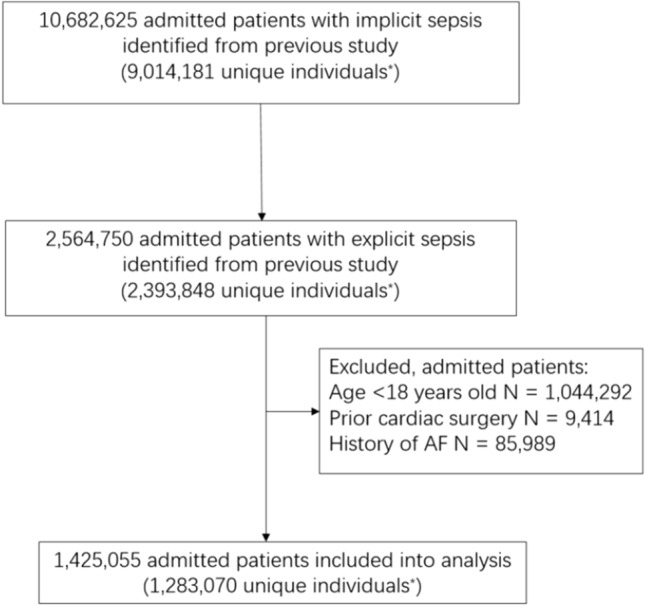
Flowchart for patients’ inclusion/exclusion, *One individual can be admitted into hospital multiple times, AF: atrial fibrillation.

After applying inclusion and exclusion criteria, a total of 1,425,055 admitted patients with explicit sepsis were included in our analysis, the flowchart was shown in Fig. 1. The median age was 67 years old and 40% of them were women. Baseline characteristics were shown in Table S10. The majority had lower respiratory tract infection (66%), followed by abdominal infection (23%) and blood stream infection (21%). The first three most common comorbidities were hypertension (31%), diabetes (21%) and congestive heart failure (19%). Presence of cardiovascular dysfunction, hematology dysfunction and use of ventilation were frequent, accounting for 27%, 10% and 9% of patients, respectively.

### Risk factors of new-onset AF

Among 1,425,055 admitted patients, 21,327 developed AF during hospitalization, with an overall incidence rate of 1.49% (95% CI 1.47, 1.51). When stratified by age, the incidence rate of new-onset AF (2.98 [95% CI 2.92, 3.04]) for patients aged more than 80 years old was almost six times higher than that (0.55 [95% CI 0.53, 0.57]) of patients aged between 18 and 65 years old. And patients with diabetes, or septic shock had slightly higher incidence risk of AF than patients without these conditions, while patients with hypertension had an incidence rate about twice than non-hypertension or non-lower respiratory infection patients (Table 1).

**Table 1:** Incidence rate of new-onset AF among the whole included patients and across different strata.

Population	Incidence rate [95% CI], %
Overall	1.49 [1.47, 1.51]
Age	
18–65 years old	0.55 [0.53, 0.57]
65–80 years old	1.76 [1.72, 1.79]
> 80 years old	2.98 [2.92, 3.04]
Diabetes	
Presence	1.68 [1.64, 1.73]
Absence	1.45 [1.42, 1.47]
Hypertension	
Presence	2.09 [2.05, 2.13]
Absence	1.24 [1.21, 1.26]
Septic shock	
Presence	1.68 [1.64, 1.73]
Absence	1.44 [1.41, 1.46]
Lower respiratory infection	
Presence	1.83 [1.81, 1.86]
Absence	0.85 [0.82, 0.88]

**Figure 2:**
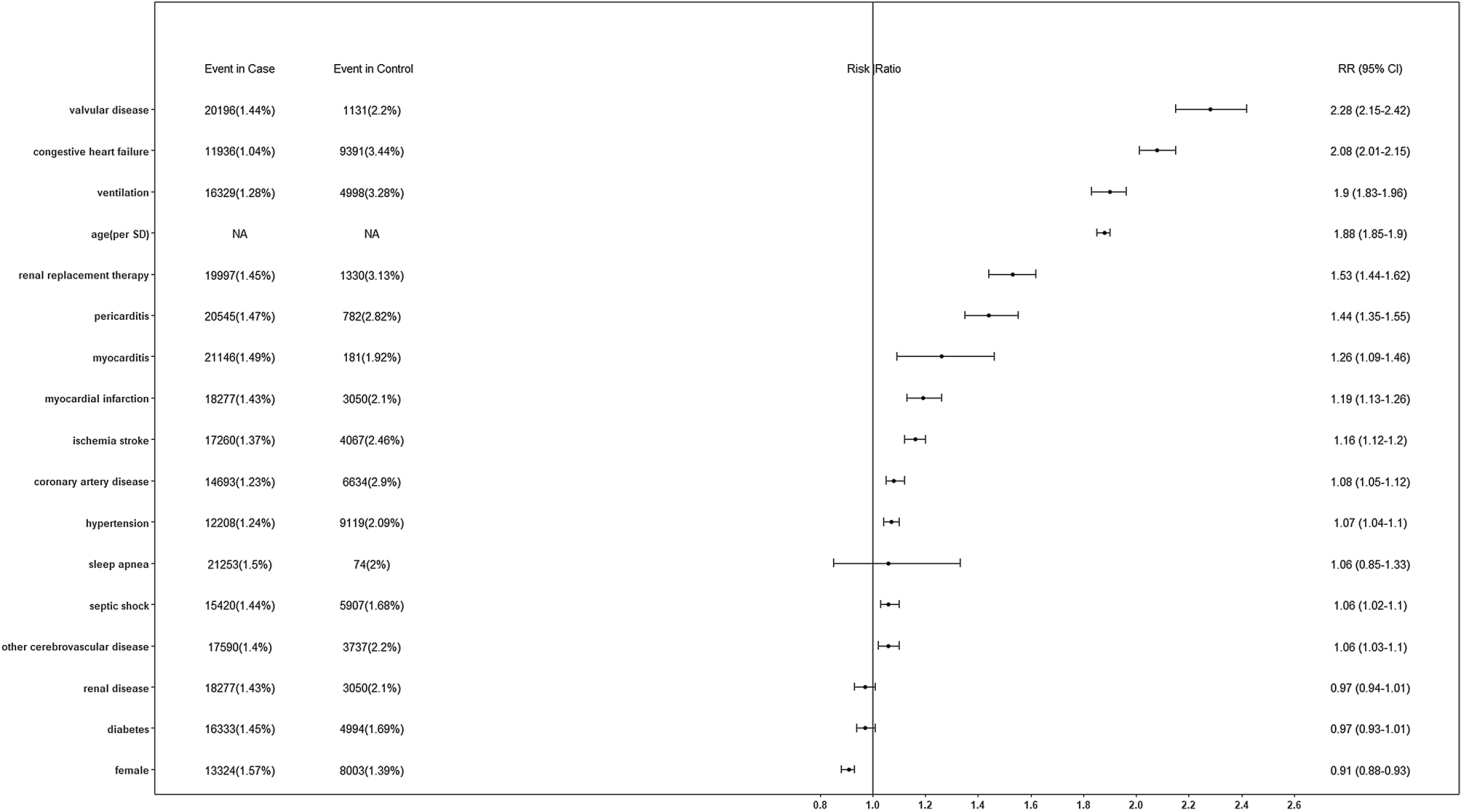
Forest plots depicting baseline factors associated with the risk of new-onset AF. Age was standardized as per standard deviation increase.

The multivariable-adjusted risk of new-onset AF associated with baseline predictors is shown in Fig. 2, factors associated with the risk of AF were older age, male sex, presence of hypertension, congestive heart failure, myocardial infarction, coronary artery disease, pericarditis, myocarditis, valvular disease, ischemic stroke, other cerebrovascular disease, septic shock, renal replacement therapy and ventilation. Their relative contribution is depicted graphically in Figure S2, age emerged as the largest contributor to the prediction of AF risk, followed by congestive heart failure and ventilation.

### Association between AF and in-hospital death

A total of 177,412 in-hospital deaths were recorded, with 172,580 deaths occurred among non-AF patients and 4832 deaths for new-onset AF patients (Table 2). The crude mortality risk ratio for new onset AF was [RR 1.50 (95% CI 1.46, 1.54)] compared to non-AF patients. After multivariable adjustment of all covariates listed in method section, including all comorbidities, organ dysfunctions and infection sites (Tables S2–S4), new onset AF was associated with a 1.03-fold [RR 1.03 (95% CI 1.01, 1.06)] higher risk of in-hospital death, and we estimated that approximately 0.2% (95% CI − 0.2%, 0.6%) of in-hospital death cases could be attributed to AF through population attributable fraction (PAF) analysis.

**Table 2:** In-hospital mortality for new-onset atrial fibrillation.

Group	n	Event	Model	RR [95% CI]	PAF (%) [95% CI]
Non-AF	1,403,728	172,580	Crude *	1.50 [1.46, 1.54]	1.42 [0.98, 1.81]
New-onset AF	21,327	4832	Model 1#	1.21 [1.18, 1.24]	0.65[0.2, 1.11]
			Model 2 ##	1.04 [1.01, 1.06]	0.2[− 0.2, 0.6]
			Model 3 ###	1.03 [1.01, 1.06]	0.2 [− 0.2, 0.6]

*Crude; #Model 1 further adjusted by age and sex, and comorbidities: hypertension, congestive heart failure, myocardial infarction, coronary artery disease, pericarditis, myocarditis, valvular disease, ischemic stroke, other cerebrovascular disease, pulmonary embolism, chronic obstructive pulmonary disease, obstructive sleep apnea, diabetes mellitus, renal disease, cancer; ##Model 2 further adjusted by organ dysfunctions: hepatic, metabolic, hematology, CNS, cardiovascular(except septic shock), septic shock and operations: ventilation, renal replacement therapy; ###Model 3 further adjusted by infection sites: cardiovascular, lower respiratory tract, abdominal, central nervous system, soft tissue or musculoskeletal, blood stream, and genitourinary.

RR: risk ratio, AF: atrial fibrillation, PAF: population attribution fraction.

Subgroup analyses suggested no interaction of diabetes and hypertension on the association of AF and in-hospital death. However, interestingly, in patients with septic shock, AF was associated with a lower risk of in-hospital death, while in patient without septic shock, a higher risk of in-hospital death was observed. Also, genitourinary and blood stream infection sites would significant (P for interaction < 0.001) but trivial (RR was almost same between groups) influenced the new-onset AF mortality risk (Table S5).

Sensitivity analyses excluding readmitted patients still showed evaluated risk of in-hospital death associated with AF (Table S6). When logistic regression model was used as an alternative to modified Poisson regression models, similar results were observed. (Tables S7–S8). The pre-existing AF associated were significantly [RR = 1.15 (95% CI 1.13, 1.16)], higher than new-onset AF (Table S9).

## Discussion

In this national-wide study of over 1.4 million sepsis patients in China, we found that the incidence of new-onset AF was 1.49%, which is lower compared to studies from Western countries (1.5% vs. 5.9%)^10,11^. The lower incidence may be due to differences in genetic susceptibilities^12^, socioeconomic factors, or disease severity^13,14^ in the studied populations. Our cohort had less severe organ dysfunction compared to Western sepsis cohorts, possibly selecting for patients at lower risk for new-onset AF.

We identified several independent risk factors for new-onset AF in sepsis, including older age, hypertension, heart failure, coronary artery disease, valvular disease, and need for mechanical ventilation. Age was the strongest predictor, aligning with AF being a disease of aging. Heart failure and coronary artery disease predispose to structural remodeling that promotes AF. Mechanical ventilation indicates greater illness severity, a known trigger for new-onset AF^15^. We did not find diabetes reduced risk against new-onset AF, differing from some studies^9^. A potential explanation is that our cohort had fewer patients with ARDS, a key factor for AF confounding diabetes^16–18^. Also, since we were using ICD codes for AF diagnosis recognition, and given the fact that AF is not a lethal condition for sepsis patients, clinicians might disregard it as an important diagnosis for submission, this might be a limitation for all administrative level study.

New-onset AF was associated with a slight 1.03-fold higher risk of in-hospital mortality after adjusting for illness severity. This suggests AF may have a small adverse impact, but the causal relationship remains uncertain. AF could worsen outcomes by impairing cardiac output and promoting thromboembolism. However, AF may just be a marker for greater disease severity. Interestingly, AF was associated with lower mortality in patients with septic shock. This could be due to earlier deaths in more severely ill patients before developing AF or changes in catecholamine management when AF occurs^19^. In subgroup analysis, genitourinary infection would influence the mortality risk for new-onset AF, this was similar in previous study^9^, and blood stream infection would influence this risk as well. However, the overall interaction effect was trivial, the subgroup didn’t change the risk ratio largely. In sensitivity analysis, the main result didn’t change when excluded those with re-admission records, and the pre-existing AF is more severe than new-onset AF as a risk factor for mortality.

Our study has implications for research and practice. Investigation of AF treatment strategies, such as rate vs rhythm control, anticoagulation, and beta-blockers, is needed to determine if improving AF management could improve sepsis outcomes. Clinicians should have heightened vigilance for AF in sepsis patients with risk factors identified here. Future research should examine if screening protocols to detect asymptomatic AF in high-risk patients improves outcomes. As a systemic inflammation disease, sepsis is highly associated with circulating inflammatory mediators such as high-sensitivity C-reactive protein (hs-CRP), interleukin (IL)-6, IL-8, etc. These pro-inflammatory mediators have impact on gap junction modulation and connexin dysregulation, calcium handling abnormalities and atrial fibrosis, which causes atrial action potential shortening, atrial ectopic activity triggering, atrial conducting slowing, and finally AF^20^. It is plausible that some chronic baseline risk factors would have synergy for these pro-inflammatory mediators, such as hypertension or heart failure, as our study claimed. As a less lethal condition, AF might work as a biomarker reminding clinicians that the patients’ imbalanced cardiac function is currently at a dangerous level. However, whether we should consider anticoagulation in sepsis-driven AF, is relatively unknown. For example, previous evidence found out that about 1/3 patients received anti-coagulation but the ischemic stroke did not significantly differ^21^. Due to lack of more solid evidences, and given our study suggesting that new-onset AF elevated mortality risk is low, conducting anticoagulation in acute phase of sepsis might be risky.

Our study has limitations. We lacked data on AF timing, recurrent episodes, clinical variables, and treatments. Administrative data may underestimate AF incidence. Nonetheless, the study provides uniquely generalizable data on AF in sepsis patients in China. Further research on AF in sepsis leveraging richer clinical data sources would be valuable.

## Conclusion

In conclusion, in this large nationwide study of sepsis in China, we determined the incidence of and risk factors for new-onset AF, which appears associated with a small increase in mortality. Our findings highlight the need to better understand how to optimize AF detection and management in sepsis patients to potentially improve outcomes.

## Method

### Data source

This was a retrospective observational database study which complied with the Guidelines for Accurate and Transparent Health Estimates Reporting statement^22^. We obtained data of hospital admissions from the National Data Center for Medical Service (NDCMS) system, which is under the authority of the National Health Commission of the People’s Republic of China and covers 1923 tertiary and 2363 secondary hospitals from 31 provinces, municipalities, and autonomous regions by the end of 2020, accounting for 64.1% of all tertiary hospitals and 22.7% of all secondary hospitals in mainland China^23^. All admissions in tertiary and secondary hospitals of NDCMS accounted for 45.0% of all hospital admissions and 69.6% of all public hospital admissions in mainland China during the study period^23^. NCDMS included de-identified individual-level information of hospital admissions including patient demographics (date of birth, sex and ethnicity), hospital information (de-identified identifier, hospital type, province-level location), admission date, discharge date, diagnoses, procedures, and cost records. The study was approved by the institutional review board of Peking Union Medical College Hospital (S-K 1911, Chinese sepsis incidence and related risk factors analysis (Jan 2022), all procedures were followed in accordance with the ethical standards of the responsible institutional committee on human experimentation and with the Helsinki Declaration of 1975). The requirement to obtain informed consents was waived because of the retrospective design.

### Study population

During the study period from 2017 to 2019, we included all admitted patients with age over 18 years old and diagnosed with explicit sepsis from the study population of the previous study, the explicit sepsis (defined by ICD-10, detail shown in Supplementary Table S1) stands for requiring patients had at least one organ failure and infection, as our previous study mentioned^24^. Among the included patients, those with any prior cardiac surgery and history of AF before admission were further excluded (Supplementary Figure S1).

### Exposure and outcome

This study contains two complementary designs. First, we analyzed the incidence of new-onset AF and evaluated baseline potential risk factors. The outcome was thus incident AF, which was defined as a discharge diagnosis of AF (ICD-10 code: I48) without appearing in the admission diagnoses. Second, we studied the association between new-onset AF and in-hospital death. To this end, AF was considered as exposure.

### Covariates

Study covariates include age, sex, organ dysfunctions, infection sites and comorbidities.

The infection sites of sepsis patients were categorized into cardiovascular, lower respiratory tract, abdominal, central nervous system, soft tissue or musculoskeletal, blood stream, and genitourinary detailed definition was shown in Supplementary Tables S2. The information of organ dysfunction and support including hepatic failure, metabolic failure, hematology failure, central nervous system failure, cardiovascular failure. Organ support including renal replacement therapy, ventilation, detailed definition was shown in Supplementary Tables S3. Comorbidities included the presence of hypertension, congestive heart failure, myocardial infarction, coronary artery disease, pericarditis, myocarditis, valvular disease, ischemic stroke, other cerebrovascular disease, pulmonary embolism, chronic obstructive pulmonary disease, obstructive sleep apnea, diabetes mellitus, chronic kidney disease, cancer (Supplementary Tables S4).

### Statistical analysis

Values are expressed as mean [standard deviation (SD)] for continuous variables with normal distribution, median [interquartile range (IQR)] for non-normal distribution variables and percentage of the total for categorical variables. First, we reported the incidence rate of new-onset AF among the whole included patients and by all strata of age (18–65, 65–80, > 80), diabetes (presence/absence), hypertension(presence/absence) and septic shock(presence/absence). Then, we evaluated baseline predictors of AF through modified Poisson regression model^25^. As previously studies mentioned^1,10,26^, we only include related predictors in prediction model: age (per standard deviation increment), sex, hypertension, chronic heart failure, myocardial infarction, coronary artery disease, pericarditis, myocarditis, valvular disease, ischemic stroke, other cerebrovascular disease, obstructive sleep apnea, diabetes mellitus, renal disease, septic shock, renal replacement therapy, ventilation. The relative importance for each predictor was evaluated by the estimated explained relative risk (McFadden’s R^2^)^27^ and overall explainable log-likelihood (χ^2^) attributable to each predictor in the analysis of variance. Next, we considered new-onset AF as an exposure, and the risk of in-hospital risk associated with it was analyzed by modified Poisson regression model. Based on biological confounders, covariates categorized by comorbidity, organ dysfunction and infection site as previously mentioned were adjusted in this analysis. That modified Poisson regression models were chosen as the models of our main analyses was based on the following reasons, first, relative risk (RR) is more explainable and easy-understanding to clinicians, which can be directly estimated by modified Poisson regression model; second, the difference between the odds ratio (OR) estimated from logistic regression and RR are negligible only for rare outcomes, while in our case, the in-hospital mortality was about 20%, thus OR is an over-estimate of RR, leading to the potential exaggeration of AF on in-hospital death risk^28,29^. Missing data handling were describe in previous study^23^, in brief, we excluded those without age, sex and diagnosis and lost to follow-ups. We don’t know if there is any diagnosis they didn’t submit, so the missing rate for covariates can’t be achieved.

Several analyses were performed to test the robustness of our data. First, stratified analyses were performed to test the consistency of our results by age strata, diabetes, hypertension and septic shock. Second, we further excluded readmitted patients (~ 1%) from our study population to assess the impact of readmitted patients on the association between AF and in-hospital death (one patient can only die one time, including readmitted patients may underestimate the risk of AF on in-hospital mortality). Third, the weighted contributions of AF to the risk of in-hospital death were quantified with the population attributable fraction (PAF) with the following equation: $PAF=p(1-1/RR)$, where $p$ denotes the prevalence of AF among the in-hospital died patients and $RR$ denotes the estimated relative risk of AF on in-hospital death. The estimate of PAF was adjusted for the above-mentioned covariates. Fourth, we included those with pre-existing AF to show if they had a higher mortality risk. Last, we replaced the modified Poisson regression models in our main analyses with logistic regression model, to see whether our results will change a lot.

### Permission to reproduce material from other sources

We accept reproducing our material from other sources in academic usage with reasonable reference.

### Supplementary Information

Supplementary Information.

## Data Availability

Source code could be shared with reasonable request to corresponding author.
