# Supplementary material for: A nationwide study on new onset atrial fibrillation risk factors and its association with hospital mortality in sepsis patients
Source: Sci Rep. 2024 May 28;14:12206. doi: 10.1038/s41598-024-62630-x (PMC11133344; doi:10.1038/s41598-024-62630-x)
Supplement: Supplementary file 1 — Supplementary Information. [file 41598_2024_62630_MOESM1_ESM.docx]

# A nationwide study on new onset atrial fibrillation risk factors and its association with hospital mortality in sepsis patients

# Supplemental Material

[**Table S1. Explicit ICD-10-CM codes of sepsis** 2](#_Toc165033863)

[**Table S2. ICD-10-CM codes for infection sites.** 5](#_Toc165033864)

[**Table S3. ICD codes of organ dysfunctions and supports** 9](#_Toc165033865)

[**Table S4. ICD codes of related comorbidities** 10](#_Toc165033866)

[**Table S5. Subgroup analyses of association of new-onset AF with in-hospital death.** 11](#_Toc165033867)

[**Table S6. Sensitivity analysis of association of new-onset AF with in-hospital death after readmitted patients excluded.** 13](#_Toc165033868)

[**Table S7. Sensitivity analysis for new-onset atrial fibrillation risk prediction in logistic model** 14](#_Toc165033869)

[**Table S8. Sensitivity analysis in-hospital mortality for new-onset atrial fibrillation in logistic model** 15](#_Toc165033870)

[**Table S9. Sensitivity analysis in-hospital mortality for new-onset and pre-existing atrial fibrillation** 16](#_Toc165033871)

[**Table S10. Baseline characteristics** 17](#_Toc165033872)

[**Figure S1. Strength of the association between hypothesized predictors and AF according to the estimated explained relative risk (McFadden’s R^2^, in red) and overall explainable log-likelihood (χ2, in blue). Age was standardized as per standard deviation increase.** 18](#_Toc164871620)

**Table S1. Explicit ICD-10-CM codes of sepsis**

| ICD-10-CM | Codes Description^a^ |
| --- | --- |
| A01.003 | Typhoid fever sepsis |
| A02.100 | Salmonella sepsis |
| A03.900 | Shigellosis, unspecified (shock) |
| A09.005 | Septic gastroenteritis |
| A20.7 | Septicemic plague |
| A21.7 | Generalized tularemia |
| A22.7 | Anthrax sepsis |
| A24.1 | Acute and fulminating melioidosis |
| A26.7 | Erysipelothrix sepsis |
| A27.900 | Leptospirosis |
| A28.001 | Pasteurellosis |
| A28.2 | Extraintestinal yersiniosis |
| A32.7 | Listerial sepsis |
| A38.x00x012 | Scarlet fever sepsis |
| A39.2 | Acute meningococcemia |
| A39.3 | Chronic meningococcemia |
| A39.4 | Meningococcemia， unspecified |
| A39.1 | Waterhouse |
| A40 | Streptococcal sepsis |
| A41 | Other sepsis |
| A42.7 | Actinomycotic sepsis |
| A48.3 | Toxic shock syndrome |
| A54.8 | Other gonococcal infections (sepsis) |
| A93.800x001 | Other specified arthropod-borne viral fevers [Piry virus disease] |
| A98.500 | Haemorrhagic fever with renal syndrome |
| B00.7 | Herpetic septicemia |
| B37.7 | Candidal sepsis |
| B37.6 | Candidal endocarditis |
| B49 | Unspecified mycosis (Fungemia) |
| F05.901 | Delirium, unspecified (infectious) |
| F06.800x002 | Other specified mental disorders due to brain damage and dysfunction and to physical disease (biliary infection) |
| F06.800x016 | Other specified mental disorders due to brain damage and dysfunction and to physical disease (bacillary dysentery) |
| J15.903 | Bacterial pneumonia, unspecified (severe community-acquired) |
| J18.903 | Pneumonia, unspecified (severe) |
| J95.000x001 | Sepsis of tracheostomy stoma |
| K85.800x019 | Abscess of pancreas (severe) |
| K85.817 | Abscess of pancreas (other, severe) |
| O03.300x001 | Spontaneous abortion, Incomplete, with septic shock |
| O03.800x001 | Spontaneous abortion, Complete, with septic shock |
| O04.300x004 | Medical abortion, Incomplete, with septic shock |
| O04.800x001 | Medical abortion, Complete, with septic shock |
| O04.804 | Medical abortion, later complete, with septic shock |
| O08.000 | Genital tract and pelvic infection following abortion and ectopic and molar pregnancy |
| O08.003 | Septic shock following abortion and ectopic and molar pregnancy |
| O08.200x002 | Embolism following abortion and ectopic and molar pregnancy (septic) |
| O08.200x006 | Embolism following abortion and ectopic and molar pregnancy (septicopyaemic) |
| O75.3 | Other infection during labour |
| O85 | Puerperal sepsis |
| O88.300 | Obstetric pyaemic and septic embolism |
| O98.8 | Other maternal infectious and parasitic diseases complicating pregnancy, childbirth and the puerperium |
| P36 | Bacterial sepsis of newborn |
| P37.800x002 | Other specified congenital infectious and parasitic diseases |
| R57.2 | Septic shock |
| R65.1 | Systemic inflammatory response syndrome (SIRS) due to infection with organ dysfunction |
| T80.2 | Infections following infusion, transfusion and therapeutic injection |
| T81.4 | Infection following a procedure, not elsewhere classified |
| T88.000x002 | Sepsis following immunization |

^a^ Any diagnosis with the word "severe" required complications of organ dysfunction in the official 10-digit Chinese version of International Classification of Diseases, Tenth Revision (ICD-10).

**Table S2. ICD-10-CM codes for infection sites.**

| Site of infection | ICD-10-CM |
| --- | --- |
| Lower respiratory tract | A01.000x005, A02.201, A06.5, A15, A16, A19.0, A19.2, A19.8, A20.2, A21.2, A22.1, A27.900x004, A31.0, A36.201, A37.800x001, A37.900x003, A37.900x004, A37.901, A42.0, A43.0, A48.1, A52.704, A54.806, B01.2, B05.2, B05.802, B20.003, B20.004, B20.6, B22.1, B25.0, B37.1, B37.800x083, B37.803, B38.0-B38.2, B39.0-B39.2, B40.0-B40.2, B41.0, B42.0, B44.0, B44.1, B45.0, B46.0, B49.x00x001, B49.x00x011, B49.x00x020, B49.x13, B49.x14, B58.3, B59.x00, B65.902, B66.401, B67.1, B67.600x001, B67.904, B67.905, B69.800x008, B69.801, B77.801, J10.0, J11.0, J11.100x005, J12-J22, J40, J42, J44.0, J44.1, J45.900x041, J45.901, J47.x03, J67.400x002, J67.600x001, J69.0, J85, J86, J90.x00x003, J90.x02, J95.000x001, J95.002, J95.800x016, J95.800x022, J95.802, J98.400x024, J98.414, J98.800x002, J98.800x009, J98.802, O99.500x010, O99.503, O99.506, P23, P24.002, P24.101, P24.901, P35.000x001, R09.1, T81.400x009, T81.403 |
| Abdominal | A00-A09, A18.3, A18.812-A18.817, A20.800x004, A21.3, A22.2, A42.1, A42.803, A50.000x002, A51.1, A51.400x008, A52.700x007, A52.700x011, A52.705, A54.6, A54.807, A56.3, B00.802, B00.803, B01.801, B05.4, B05.800x003, B15 – B17, B19, B20.005, B20.006, B25.1, B25.2, B26.3, B26.802, B37.800x091, B37.805, B37.806, B44.803, B45.800x001, B46.2, B49.x00x002, B49.x00x021, B49.x16, B49.x17, B51.0, B54.x00x003, B57.3, B65.202, B65.903, B66.501, B66.902, B67.0, B67.5, B67.8, B67.907, B68.900x003, B69.802, B76.901, B76.902, B77.0, B77.803, B77, B78.0, B81, B82, B87.800x002, D73.3, D73.800x007, E84.1, F06.800x002, F06.800x016, I88.0, J10.800x002, J11.800x002, K25.1, K25.2, K25.5, K25.6, K26.1, K26.2, K26.5, K26.6, K27.1, K27.2, K27.5, K27.6, K28.1, K28.2, K28.5, K28.6, K31.800x806, K31.814, K35 - K37, K38.800x004, K40.0, K40.1, K40.3, K40.4, K41.0, K41.1, K41.3, K41.4, K42.0, K42.1, K43.0, K43.1, K44.0, K44.1, K45.0, K45.1, K46.0, K46.1, K50.000x001, K55.0, K55.9, K56, K57.0, K57.2, K57.304, K57.305, K57.4, K57.8, K57.900x001, K61, K62.4, K62.800, K62.801, K62.802, K62.807, K62.811, K62.814, K62.815, K62.821, K62.822, K62.900x001, K63.0, K63.1, K63.800x011, K63.817, K65, K66.800x008, K67.0-K67.2, K71.1, K75.0, K80-K85, K86.100x004, K86.811, K91.3, K91.800x106, K91.800x111, K91.800x412, K91.800x704, K91.800x705, K91.800x706, K91.802, K91.808, K91.811, K91.814, K91.821, K91.829, K91.831, K91.834, K91.837, K91.842, O26.600x010, O98.800x007, O98.810, O98.811, O99.600x001, O99.600x006, O99.600x007, O99.600x017, O99.603-O99.607, O99.610, O99.611, O99.617- O99.619, O99.621, O99.624, P38, P59.201, P59.202, P76, P77, P78.0, P78.1, P78.3, P78.300x002, P78.300x005, P78.800x007, P78.800x009, R19.500x003, T80.203, T81.203, T81.204,T81.400x010, T81.400x013, T81.404, T81.408, T81.601, T81.800x006, T85.708-T85.711 |
|  |  |
| Genitourinary | A02.200x004, A06.800x001, A06.800x003, A06.800x004, A18.1, A36.800x005, A42.804, A42.805, A43.8, A51.0, A51.304, A51.400x009, A52.700x012, A54.0-A54.2, A55, A56.0 - A56.2, A57, A58, B01.800x002, B26.0, B26.800x008, B26.800x009, B37.3, B37.4, B45.800x002, B49.x18, B65.000x002, B65.001, B87.800x001, N13.6, N15.1, N15.9, N18.800x014, N28.800x005, N28.800x007, N30.0, N30.801, N34.0, N34.204, N37.0, N39.0, N41.0, N41.2, N41.900x002, N43.1, N45, N48.2, N48.800x005, N49.102, N49.2, N51.2, N70.9, N71.0, N71.9, N72, N73.0-N73.3, N73.5, N73.8, N73.9, N74.2, N74.3, N74.8, N75.1, N76.0, N76.2, N76.4, N77.1, N77.8, N98.0, O03.0, O03.5, O04.0, O04.5, O05.0, O05.5, O06.0, O06.5, O07.0, O07.5, O08.0, O23, O75.300x002, O86.0, O86.1, O86.2, O86.3, O86.800x001, O86.802, O98.3, O98.800x002, O98.800x013, O98.806, O98.808, P39.3, Q61.300x002, T81.205, T81.400x014, T81.409, T83.5, T83.6 |
|  |  |
| Nervous system | A01.000x003, A02.203, A03.900x008, A06.600, A17, A18.800x043, A20.3, A22.801, A23.900x005, A23.900x006, A27.900x006, A32.1, A32.801, A36.803, A37.900x005, A39.0, A39.1, A39.802, A39.803, A39.9, A42.800x003, A43.801, A48.100x003, A50.4, A51.401, A52.1 - A52.3, A54.801, A54.803, A69.2, A80 - A89, A92.300x002 - A92.300x004, B00.3, B00.4, B01.0, B01.1, B02.0 - B02.2, B05.0, B05.1, B06.0, B22.0, B25.801, B26.1, B26.2, B26.800x003, B37.5, B37.800x085, B38.4, B43.1, B44.800x005, B45.1, B49.x00x013, B49.x01, B50.0, B57.4, B58.2, B60.2, B65.901, B66.901, B67.600x002, B67.902, B68.900x002, B69.0, B69.800x005, B83.200x003, B83.202, B89.x01, F06.803, G00, G01, G02, G03.002, G03.2, G03.800, G03.900-G03.904, G03.907, G04.000, G04.001, G04.2, G04.8, G04.9, G05, G06.0, G06.1, G06.2, G07, G08.x00x001 - G08.x00x003, G08.x00x006, G08.x00x009, G08.x01, G91.000x003, G97.100x002, I60.900x005, I67.100x012, I68.1, I68.2, J10.800x001, J10.800x012, J11.800x001, O98.501, P37.800x001, P39.801, T85.701, T85.702 |
|  |  |
| Soft tissue or musculoskeletal | A02.200x005, A02.202, A06.700, A18.0, A18.4, A18.800x010, A18.800x022, A18.800x027, A18.800x028, A18.810, A18.819, A20.100, A20.101, A23.901, A23.902, A31.100, A32.000, A39.804, A42.800x002, A43.100, A44.100, A46.x00, A48.0, A51.300-A51.303, A51.400x002, A51.400x007, A52.103, A52.700x008, A52.700x009, A52.700x010, A52.700x023, A52.706, A54.401-A54.405, A54.800x002, A69.900x002, B26.800x001, B33.0, B35.801, B37.2, B37.800x089, B37.800x090, B38.3, B40.301, B40.302, B41.800x002, B42.1, B43.0 B43.2, B45.2, B45.3, B46.3, B47.0, B47.100x001, B49.x00x027, B58.800x002, B65.3, B66.400x001, B67.2, B67.600x003, B69.800x003, B69.800x004, B69.803 - B69.805, B76.900x003, B78.1, B88.000x004, B88.000x006, B88.001, B88.800x004, B88.900x001, B88.900x003, B89.x00x002, E10.500, E10.505, E10.600x911, E11.500, E11.505, E11.600x911, E14.500x021, E14.500x042, E14.500x044 - E14.500x062, E14.600x911, E16.800x021, I70.200x041, I70.200x072, I70.200x073, I70.202, I70.205, I70.207, I70.800x012, I70.900x011, I70.900x012, J98.500x001, J98.501-J98.503, K04.0, K04.1, K04.4-K04.7, K05.0-K05.2, K05.3, K05.500x002, K06.801, K07.604, K10.200, K10.202-K10.205, K10.207-K10.210, K10.301, K11.200-K11.203, K11.208-K11.211, K11.3, K12.001, K12.200-K12.204, K12.210-K12.215, K12.217, K12.218, K13.000x001, K13.000x016, K13.011, K13.400x004, K14.001, K22.800x021, L00, L02-L03, L05, L08, L30.3, L73.2, L73.800x006, L73.800x007, L84, L88, M00, M01, M03, M31.200x003, M31.200x011, M46.3, M46.5, M46.801, M49.1, M49.2, M60.0, M63.0-M63.2, M65.0, M65.1, M68.0, M71.0, M71.1, M72.8, M79.800x081, M86, M90.1, M90.6, M93.901, M93.902, M94.801, M94.805, N61, O91, O99.700x006, O99.704, P39.4, P83.802, T79.3, T81.400x001, T81.400x011, T81.400x012, T81.405 - T81.407, T84.5 - T84.7, T85.712, T85.713, T86.807, T87.001, T87.101, T87.400, T98.200x012, T98.200x021 |
|  |  |
| Cardiovascular | A01.000x016, A18.800x002, A18.800x017, A18.800x018, A18.800x031, A18.800x042, A18.808, A18.809, A18.818, A32.802, A36.802, A38.x00x002, A39.5, A52.0, A54.802, A54.804, A54.805, B01.800x001, B05.803, B25.803, B26.803, B33.2, B37.6, B49.x15, B57.0, B58.800x001, B67.903, I01, I09, I26.900x017, I26.900x018, I30.1, I30.8, I30.9, I31.9, I32.0, I32.1, I32.8, I33.0, I33.9, I34.800x005, I35.100x012, I35.101, I35.800x003, I35.803, I35.807, I38, I39.8, I40, I41.0-I41.2, I41.8, I51.4, I51.802, I52.0, I70.901, I71.900x004, I77.604, I77.605, I77.806, I79.1, I80, I82.1, I83.1, J10.800x003, J10.802, J11.800x003, J11.800x011, O99.400x031, T81.202, T81.702, T82.6, T82.7 |
| Blood stream | A39.2-A39.4, A49.001, A49.101, A49.201, A49.301, A49.803, A49.806, A49.811, A49.813, A49.901, B25.800x001, B34.900x002, O98.804, P36.902 |

**Table S3. ICD codes of organ dysfunctions and supports**

| **System** | **Diagnosis (ICD-10) or**  **Procedure (ICD-9-CM)** | **Code** |
| --- | --- | --- |
| Cardiovascular (except septic shock) related diagnosis | Diagnosis (ICD-10) | A48.3, E86.x00, E86.x00x001, E86.x00x003, E86.x00x004, E86.x00x005, E86.x01, I51.400x007, I95.8, I95.9, I99.x00, I99.x01, R09.800x082, R57.0, R57.1, R57.8, R57.9, P29 |
|  | Procedure (ICD-9-CM) | 3893, 3897, 3899 |
| Ventilation related procedures | Procedure (ICD-9-CM) | 9390, 9391, 9604, 9670, 9671, 9672 |
| Central nervous system related diagnosis | Diagnosis (ICD-10) | F05, F06.8, G93.1, G93.4, G93.8, G93.9, R40, R41.0, R45.3, R55 |
| Renal replacement therapy procedures | Procedure (ICD-9-CM) | 3895, 3927, 3942, 3995, 5498, 3895 |
| Metabolic related diagnosis | Diagnosis (ICD-10) | E87.2 |
| Hematologic related diagnosis | Diagnosis (ICD-10) | A93.800x001, D61.900x001, D61.901, D61.903, D61.906, D65, D65.x00x003, D65.x01, D65.x02, D65.x03, D68.9, D69.000x008, D69.000x011, D69.000x013, D69.203, D69.301, D69.5, D69.501, D69.6, D69.8, D76.200x001, D76.200x011 |
| Hepatic related diagnosis | Diagnosis (ICD-10) | B15.000, B15.001, B15.002, B15.003, B16.000, B16.001, B16.200, B16.201, B16.202, B16.203, B16.204, B16.206, B17.807, B19.000, B19.000x001, B19.001, B19.002, B25.101, E80.600, E80.604, E80.700, K71.100x001, K71.103, K72.0, K72.9, K76.7, K76.8, K76.9, K91.825 |
| Hepatic related procedures | Procedure (ICD-9-CM) | B15.000, B15.001, B15.002, B15.003, B16.000, B16.001, B16.200, B16.201, B16.202, B16.203, B16.204, B16.206, B17.807, B19.000, B19.000x001, B19.001, B19.002, B25.101, E80.600, E80.604, E80.700, K71.100x001, K71.103, K72.0, K72.9, K76.7, K76.8, K76.9, K91.825 |
| Septic shock | Diagnosis (ICD-10) | A03.900x007, A41.900x003, A48.3, O03.300x001, O03.800x001, O04.300x004, O04.800x001, O04.804, O08.003, R57.2, R57.800x003, T80.200x003 |

**Table S4. ICD codes of related comorbidities**

| **Comorbidity** | **ICD-10 Code** |
| --- | --- |
| Hypertension | I10-I13, I15 |
| Heart failure | I11.0, I50, I97.1 |
| Myocardial infarction | I21, I22, I25.2 |
| Coronary artery disease | I20, I23, I24, I25.1, I25.3-I25.9 |
| Pericarditis | A39.53, B33.23, I01.0, I09.2, I30.0, I30.1, I30.8, I30.9, I31, I32 |
| Myocarditis | I40.0, I40.1, I40.8, I40.9, I41, I51.4, J10.82, J11.82, A38.1, A39.52, B26.82, B33.22, B58.81, D86.85, I01.2, I09.0 |
| Valvular Disease | I05-I09, I34-I36 |
| Ischemic stroke | I63, I64 |
| Other cerebrovascular disease | I65-I69, G45.4, G46 |
| Pneumonia | A15, A16, A37.00, A37.01, A37.10, A37.11, A37.80, A37.81, A37.90, A37.91, A54.84, B37.1, B38.0-B38.2, B39.0-B39.2, B40.0-B40.2, B41.0, B41.0, B44, B45.0, B46.0, B49.0, B49.x00, B49.x14, B65.902, B66.401, B67.1, B67.600x001, B67.904, B67.905, B69.801, J10.0, J11.0, J12.0-J18, J44, J47.x03, J69, J85, J95.802, J98.400x024, J98.414, J99.8 |
| Pulmonary embolism | I26.01, I26.02, I26.09, I26.90, I26.92, I26.99 |
| Chronic obstructive pulmonary disease | J43, J44.00, J44.01, J44.09 |
| Obstructive sleep apnea | G47.3 |
| Diabetes mellitus | E10-E14 |
| Renal disease | I12.0, I13.1, N03.2-N03.7, N05.2-N05.7, N18, N19, N25.0, Z49.0-Z49.2, Z94.0, Z99.2 |
| Cancer | C00-C97 |

**Table S5. Subgroup analyses** **of association of new-onset AF with in-hospital death.**

| Group | RR [95%CI] | P for interaction |
| --- | --- | --- |
| Age strata |  |  |
| 18-65 | 1.10 [1.03, 1.18] |  |
| 65-80  >80 | 1.10 [1.06, 1.15]  1.13 [1.10, 1.17] | 0.33  0.08 |
| Diabetes |  |  |
| Presence | 1.09 [1.07,1.12] | 0.44 |
| Absence | 1.11 [1.09,1.14] |  |
| Hypertension |  |  |
| Presence | 1.11 [1.07, 1.15] | 0.35 |
| Absence | 1.10 [1.07, 1.14] |  |
| Septic Shock |  |  |
| Presence | 0.92 [0.90,0.95] | <0.001 |
| Absence | 1.17 [1.14,1.20] |  |
| Lower respiratory infection | |  |
| Presence | 1.10 [1.07,1.13] | 0.04 |
| Absence | 1.09 [1.00,1.19] |  |
| Cardiovascular infection | |  |
| Presence | 1.20 [0.99,1.44] | 0.8 |
| Absence | 1.10 [1.07,1.14] |  |
| Genitourinary infection | |  |
| Presence | 1.12 [1.04,1.21] | <0.001 |
| Absence | 1.10 [1.07,1.13] |  |
| Abdominal infection | |  |
| Presence | 1.09 [1.03, 1.15] | 0.08 |
| Absence | 1.11 [1.08, 1.15] |  |
| Central nervous system infection | |  |
| Presence | 1.57 [1.26, 1.96] | 0.7 |
| Absence | 1.10 [1.07, 1.13] |  |
| Soft tissue or musculoskeletal infection | |  |
| Presence | 1.10 [0.98, 1.24] | 0.4 |
| Absence | 1.10 [1.07, 1.13] |  |
| Blood stream infection | |  |
| Presence | 1.19 [1.08, 1.31] | <0.001 |
| Absence | 1.09 [1.06, 1.12] |  |

All model adjusted by age, sex; comorbidities: hypertension, chronic heart failure, myocardial infarction, coronary artery disease, pericarditis, myocarditis, valvular disease, ischemic stroke, other cerebrovascular disease, pneumonia, pulmonary embolism, chronic obstructive pulmonary disease, obstructive sleep apnea, diabetes mellitus, renal disease, cancer; organ dysfunctions: hepatic, metabolic, hematology, CNS, cardiovascular (except septic shock), septic shock; operations: ventilation, renal replacement therapy; infection sites: cardiovascular, lower respiratory tract, abdominal, central nervous system, soft tissue or musculoskeletal, blood stream, and genitourinary; Certain covariate would be excluded for certain subgroup, e.g. diabetes would be excluded for diabetes subgroup analysis.

AF: atrial fibrillation, RR: risk ratio.

**Table S6. Sensitivity analysis of association of new-onset AF with in-hospital death after readmitted patients excluded.**

| Group | n | Event | Model | RR (95%CI) |
| --- | --- | --- | --- | --- |
| Control | 1,263,072 | 156,064 | Crude* | 1.65 [1.60, 1.71] |
| AF | 19,998 | 4,499 | Model1# | 1.32 [1.27,1.36] |
|  |  |  | Model2## | 1.08 [1.05,1.12] |
|  |  |  | Model3### | 1.02 [1.00, 1.04] |

*Crude; #Model 1 adjusted by age and sex, further adjusted by comorbidities: hypertension, chronic heart failure, myocardial infarction, coronary artery disease, pericarditis, myocarditis, valvular disease, ischemic stroke, other cerebrovascular disease, pulmonary embolism, chronic obstructive pulmonary disease, obstructive sleep apnea, diabetes mellitus, renal disease, cancer; ##Model 2 further adjusted by organ dysfunctions: hepatic, metabolic, hematology, CNS, cardiovascular (except septic shock), septic shock and operations: ventilation, renal replacement therapy; ###Model 3 further adjusted by infection sites: cardiovascular, lower respiratory tract, abdominal, central nervous system, soft tissue or musculoskeletal, blood stream, and genitourinary

RR: risk ratio, AF: atrial fibrillation

**Table S7. Sensitivity analysis for new-onset atrial fibrillation risk prediction in logistic model**

| Covariates | OR [95%CI] |
| --- | --- |
| Age | 1.04 [1.04, 1.04] |
| Female | 0.90 [0.88, 0.93] |
| Hypertension | 1.07 [1.04, 1.10] |
| CHF | 2.12 [2.06, 2.19] |
| MI | 1.21 [1.14, 1.28] |
| CAD | 1.09 [1.05, 1.13] |
| Pericarditis | 1.47 [1.36, 1.58] |
| Myocarditis | 1.27 [1.09, 1.47] |
| Valvular disease | 2.40 [2.25, 2.56] |
| Ischemic stroke | 1.16 [1.12, 1.21] |
| Other cerebrovascular disease | 1.06 [1.02, 1.10] |
| Obstructive sleep apnea | 1.07 [0.84, 1.35] |
| Diabetes mellitus | 0.97 [0.94, 1.01] |
| Renal disease | 0.97 [0.93, 1.01] |
| Septic shock | 1.07 [1.03, 1.10] |
| RRT | 1.56 [1.47, 1.66] |
| Ventilation | 1.94 [1.88, 2.01] |

CHF: Chronic Heart failure, RRT: Renal replacement therapy, MI: Myocardial infarction, CAD: Coronary artery disease

**Table S8. Sensitivity analysis in-hospital mortality for new-onset atrial fibrillation in logistic model**

| Group | n | Event | Model | OR [95%CI] |
| --- | --- | --- | --- | --- |
| Control | 1,403,728 | 172,580 | Crude* | 1.65 [1.60, 1.71] |
| AF | 21,327 | 4,832 | Model 1# | 1.32 [1.27,1.36] |
|  |  |  | Model 2## | 1.08 [1.05,1.12] |
|  |  |  | Model 3### | 1.07 [1.04, 1.11] |

*Crude; #Model 1 adjusted by age and sex, further adjusted by comorbidities: hypertension, chronic heart failure, myocardial infarction, coronary artery disease, pericarditis, myocarditis, valvular disease, ischemic stroke, other cerebrovascular disease, pulmonary embolism, chronic obstructive pulmonary disease, obstructive sleep apnea, diabetes mellitus, renal disease, cancer; ##Model 2 further adjusted by organ dysfunctions: hepatic, metabolic, hematology, CNS, cardiovascular (except septic shock), septic shock and operations: ventilation, renal replacement therapy; ###Model 3 further adjusted by infection sites: cardiovascular, lower respiratory tract, abdominal, central nervous system, soft tissue or musculoskeletal, blood stream, and genitourinary

OR: odds ratio, AF: atrial fibrillation

**Table S9. Sensitivity analysis in-hospital mortality for new-onset and pre-existing atrial fibrillation**

| Group | n | Event | RR [95%CI] |
| --- | --- | --- | --- |
| Control | 1,403,728 | 172,580 | 1 (Ref) |
| New-onset AF# | 21,327 | 4,832 | 1.03 [1.01, 1.06] |
| Pre-existing AF# | 85,766 | 19,661 | 1.15 [1.13,1.16] |

*Crude; #Model adjusted by age and sex, and comorbidities: hypertension, chronic heart failure, myocardial infarction, coronary artery disease, pericarditis, myocarditis, valvular disease, ischemic stroke, other cerebrovascular disease, pulmonary embolism, chronic obstructive pulmonary disease, obstructive sleep apnea, diabetes mellitus, renal disease, cancer; organ dysfunctions: hepatic, metabolic, hematology, CNS, cardiovascular (except septic shock), septic shock and operations: ventilation, renal replacement therapy; as well as infection sites: cardiovascular, lower respiratory tract, abdominal, central nervous system, soft tissue or musculoskeletal, blood stream, and genitourinary

RR: risk ratio, AF: atrial fibrillation

**Table S10. Baseline characteristics**

| Covariates | All included patients | Patients with new-onset AF |
| --- | --- | --- |
| n | 1,425,055 | 21,327 |
| Age Median [IQR] | 67 [54,78] | 78 [69,84] |
| Age category, n (%) |  |  |
| 18-65 | 624,067(44) | 3,443(16) |
| 65-80 | 489,671(34) | 8,600(40) |
| >80 | 311,317(22) | 9,284(44) |
| Female (n(%)) | 575,455(40) | 8,003(38) |
| **Infection sites**, n (%) |  |  |
| Lower respiratory tract | 937,110(66) | 17,186(81) |
| Abdominal | 329,513(23) | 5,355(25) |
| Genitourinary | 168,762(12) | 2,625(12) |
| Central nervous system | 21,017(2) | 229(1) |
| Soft tissue or musculoskeletal | 60,334(4) | 1,002(5) |
| Cardiovascular | 17,918(1) | 413(2) |
| Blood stream | 290,336(21) | 3,108(15) |
| **Comorbidities**, n (%) |  |  |
| Hypertension | 436,871(31) | 9,119(43) |
| Congestive heart failure | 272,789(19) | 9,391(44) |
| Myocardial infarction | 35,490(2.5) | 1,337(6.3) |
| Coronary artery disease | 228,673(16) | 6,634(31) |
| Pericarditis | 27,738(2) | 782(4) |
| Myocarditis | 9,433(1) | 782(4) |
| Valvular Disease | 21,296(2) | 1,131(5) |
| Ischemic stroke | 165,120(12) | 4,067(19) |
| Other cerebrovascular disease | 169,715(12) | 3,737(18) |
| Pulmonary embolism | 12,941(1) | 333(2) |
| Chronic obstructive pulmonary disease | 173,303(12) | 3,444(15) |
| Obstructive sleep apnea | 3,708(0.3) | 74(0.3) |
| Diabetes mellitus | 296,159(21) | 4,994(23) |
| Renal disease | 145,538(11) | 3,050(14) |
| Cancer | 167,550(12) | 2,484(12) |
| **Organ dysfunctions and supports**, n (%) |  |  |
| Ventilation | 152,276(11) | 4,998(23) |
| Renal replacement therapy | 42,513(3) | 1330(6) |
| Cardiovascular Dysfunction (except septic shock) | 180,019(13) | 5,848(27) |
| Hepatic Dysfunction | 132,155(9) | 2,724(13) |
| Metabolic Dysfunction | 110,439(8) | 3,035(14) |
| Hematology Dysfunction | 141,241(10) | 3,881(18) |
| Central nervous system Dysfunction | 46,185(3) | 1,041(5) |
| Septic shock | 351,155(25) | 5,907(28) |


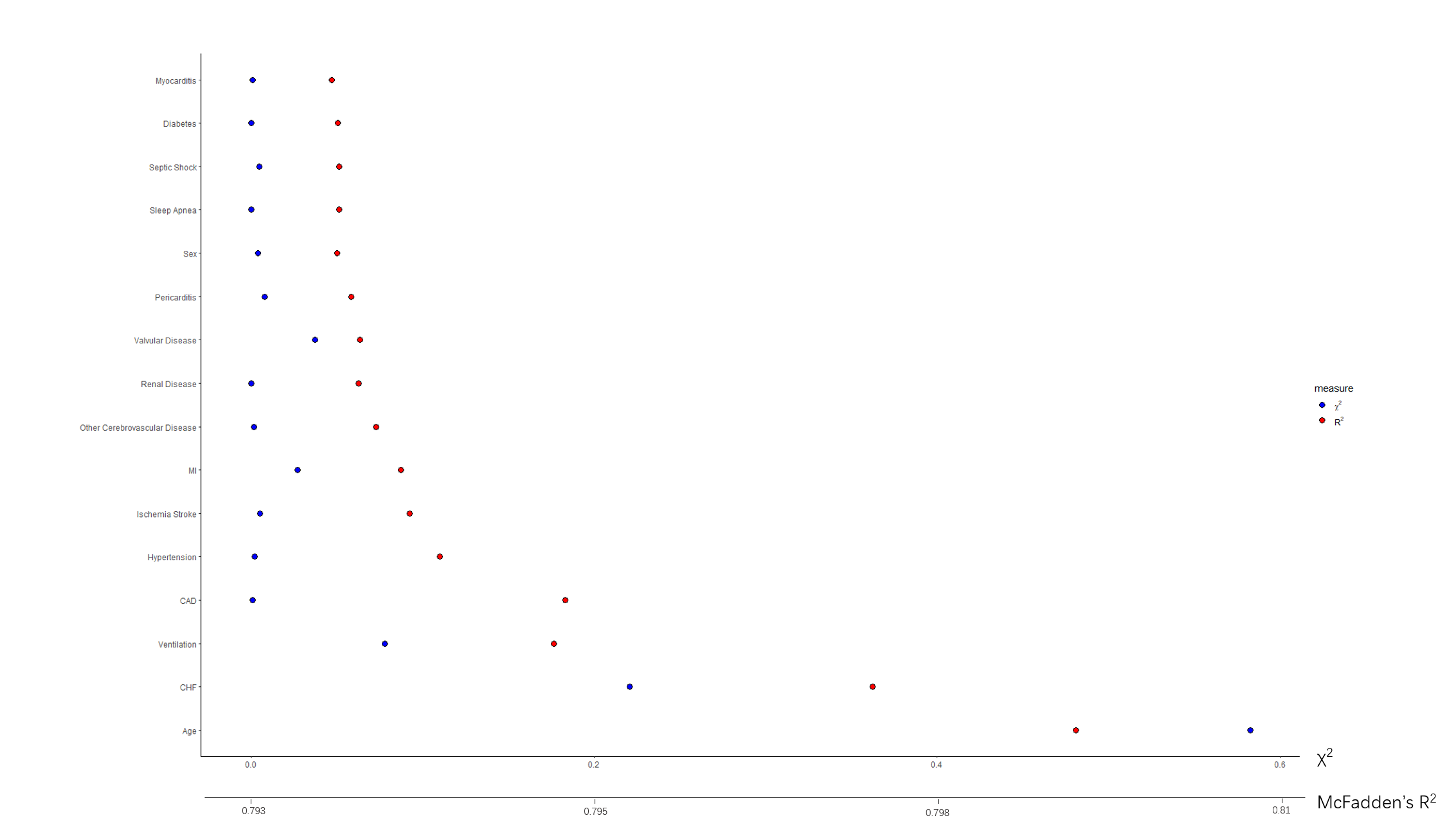


**Figure S1. Strength of the association between hypothesized predictors and AF according to the estimated explained relative risk (McFadden’s R^2^, in red) and overall explainable log-likelihood (χ2, in blue). Age was standardized as per standard deviation increase.**

MI: myocardial infarction, CAD: coronary artery disease, CHF: chronic heart failure
